# Possible Implication of Nrf2, PPAR-γ and MAPKs Signaling in the Protective Role of Mangiferin against Renal Ischemia/Reperfusion in Rats

**DOI:** 10.3390/ph16010006

**Published:** 2022-12-21

**Authors:** Abdallah M. Gendy, Amira A. El-Gazar, Ghada M. Ragab, Asmaa K. Al-Mokaddem, Alaadin E. El-Haddad, Heba Mohammed Refat M. Selim, Einas Mohamed Yousef, Najat O. Hamed, Sherihan Salaheldin Abdelhamid Ibrahim

**Affiliations:** 1 Pharmacology and Toxicology Department, Faculty of Pharmacy, October 6 University, Giza 12585, Egypt; 2 Pharmacology and Toxicology Department, Faculty of Pharmacy, Misr University for Science and Technology, Giza 12585, Egypt; 3 Pathology Department, Faculty of Veterinary Medicine, Cairo University, Giza 12211, Egypt; 4 Pharmacognosy Department, Faculty of Pharmacy, October 6 University, Giza 12585, Egypt; 5 Pharmaceutical Sciences Department, Faculty of Pharmacy, Al-Maarefa University, Diriyah, Riyadh 13713, Saudi Arabia; 6 Microbiology and Immunology Department, Faculty of Pharmacy (Girls), Al-Azhar University, Cairo 12463, Egypt; 7 Histology and Cell biology Department, Faculty of Medicine, Menoufia University, Menoufia 11411, Egypt; 8 Pharmacology and Therapeutics Department, Faculty of Pharmacy, Pharos University in Alexandria, Alexandria 21523, Egypt

**Keywords:** mangiferin, renal ischemia, Nrf2/HO-1, PPAR-γ/NF-κB, MAPK/JNK

## Abstract

Mangiferin (Mang) is a known glucosylxanthone that has proven its shielding effect against ischemia/reperfusion (Is/R). However, its full underlying mechanistic perspective against renal Is/R induced lesions is not fully revealed. Consequently, the purpose of this study is to track further non-investigated modulatory signals of Mang against the renal Is/R model involving nuclear factor erythroid 2-related factor (Nrf)2/heme oxygenase (HO)-1, peroxisome proliferator-activated receptor (PPAR)-γ/nuclear factor (NF)-κB, p38 mitogen-activated protein kinase (MAPK), and c-Jun N-terminal kinase (JNK) signaling. To ratify our aim, Mang was administrated (20 mg/kg, i.p for seven days) before the induction of bilateral Is/R. Mechanistic maneuver revealed that Mang balanced oxidative state via increasing the expression of the antioxidant Nrf2/HO-1 cue with subsequent enhancement of GSH besides MDA lessening. Additionally, Mang enhanced PPAR-γ mRNA expression and declined *p*-p38 MAPK and *p*-JNK expression with concomitant NF-κB downsizing leading to iNOS/NOx and TNF-α rebating. Furthermore, the Mang anti-apoptotic trait was affirmed by enriching Bcl-2 expression as well as decreasing Bax and caspase-3 expression. All these potentials were in the line with the molecular docking results and the improved histopathological findings and renal function biomarkers. Consequently, Mang provided plausible protective mechanisms against renal Is/R-related events, possibly by amending oxidative status, inflammatory mediators, and apoptotic cell death through the involvement of Nrf2, PPAR-γ, MAPK, JNK, and NF-κB signaling.

## 1. Introduction

Acute kidney injury (AKI) is a global serious pathological condition that is accompanied by a decline in renal output with long-term care and a high mortality rate [1]. One of the leading AKI causes is ischemia/reperfusion (Is/R) injury. Renal Is/R is a frequent consequence of renal transplantation, partial nephrectomy, and shock during resection of renal tumors [2,3]. 

The renal Is/R archetype involves various players that contribute to the negative sequences of Is/R lesions, such as oxidative stress (os), inflammatory condition/pro-inflammatory cytokines release, and apoptosis [4]. One of the assorted elements acting as a shelter against os lesion, attenuating the inflammatory cascades implicated in renal Is/R injury is the transcriptional factor nuclear factor erythroid-2-related factor-2 (Nrf2) which is a rescue signal to counteract os [5]. The Nrf2 is shifted into the nucleus after Is/R departs its cytoplasmic repressor triggering one of the crucial detoxifying genes, the inducible heme oxygenase-1 (HO-1) [4,6]. The latter interacts with several ischemic stressors and acts as a protective stress-responsive protein.

On the other hand, the peroxisome proliferator-activated receptor gamma (PPAR-γ) agonists have been previously distinguished as possessing beneficial outcomes in renal Is/R conditions [7]. The PPAR-γ modulators effect was related mainly to attenuating the Is/R induced changes, namely, apoptosis and nuclear factor-kappa B (NF-κB) expression along with its downstream target genes such as pro-inflammatory cytokines [8]. Moreover, the characters of p38 mitogen-activated protein kinase (p38 MAPK) and c-Jun N-terminal kinase (JNK) have been studied previously in inducing renal Is/R injury via activating NF-κB, causing an intensification in the transcription of inflammatory and apoptotic mediators. The modulation of the aforementioned pathways suppressed the renal Is/R injury in rats [9,10].

Cell death by apoptosis has played a fundamental part in the pathogenesis of renal Is/R lesions manifested by the enhancement of Bcl-2-associated X protein (Bax), as well as capsase-3 and the reduction of B-cell lymphoma-2 (Bcl-2) [11]. 

Mangiferin (Mang) is a natural xanthone isolated mainly from mango leaves (*Mangifera indica* Linn) that has shown various beneficial pharmacological effects including anti-inflammatory, anti-apoptosis, antioxidant, anti-cancer, and neuroprotective activities [12,13]. Previously, Wang et al. [14] have reported the Mang protective effect against renal Is/R, however, its molecular targets entailing its reno-protective effect have not been fully studied. The current research evaluated the modulatory effect of Mang on the Nrf2/HO-1, PPAR-γ/NF-κB, p38 MAPK, and JNK trajectories in the renal Is/R model.

## 2. Results

It is noteworthy to mention that group 2 (Mang administration without Is/R induction) does not show a significant difference from the sham (Sh) group.

### 2.1. Impact of Mang on the Expression of Nrf2 and HO-1 Genes

**Figure 1:**
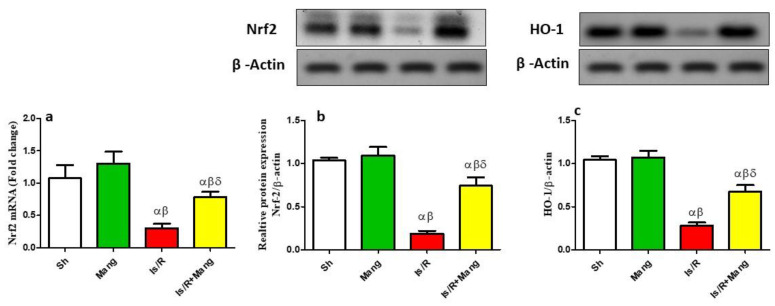
Mang’s (20 mg/kg, i.p) impact on renal Nrf2 either the (**a**) mRNA or (**b**) protein expression and (**c**) HO-1 protein expression as an antioxidant signaling cue. Data were articulated as the mean (*n* = 7) ± SD, *p* value < 0.05 is significant. As compared with sham (α), Mang (β), and Is/R (δ) treated groups.

As illuminated in Figure 1, Is/R injury caused a down-regulation in the mRNA and protein expression of Nrf2 as well as HO-1 protein expression. Within the Mang pretreatment schedule (group 4), a spike of these genes was more lucent, elucidating Mang’s antioxidant nature (Western supplementary as Figure S1a,b). 

### 2.2. Impact of Mang on Oxidative Stress Biomarkers

**Figure 2:**
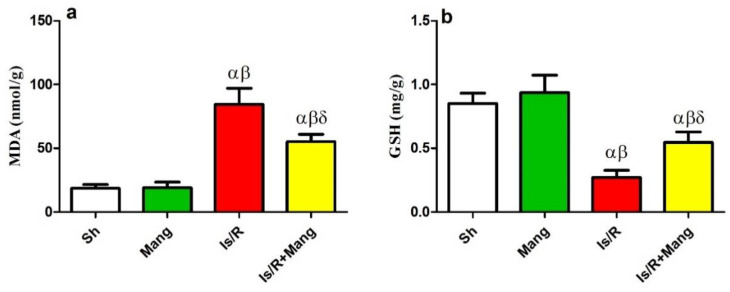
Mang’s (20 mg/kg, i.p) impact on renal (**a**) MDA and (**b**) GSH contents as oxidative stress markers. Data were articulated as the mean (*n* = 7) ± SD, *p* value < 0.05 is significant. As compared with sham (α), Mang (β), and Is/R (δ) treated groups.

Renal Is/R provokes os environment revealed by renal malondialdehyde (MDA) building up (451%) and lessening of glutathione (GSH; 32%), compared to Sh. On the contrary, Mang opposed these alterations in both biomarkers (Figure 2).

### 2.3. Impact of Mang on Nitrosative Stress Parameters

**Figure 3:**
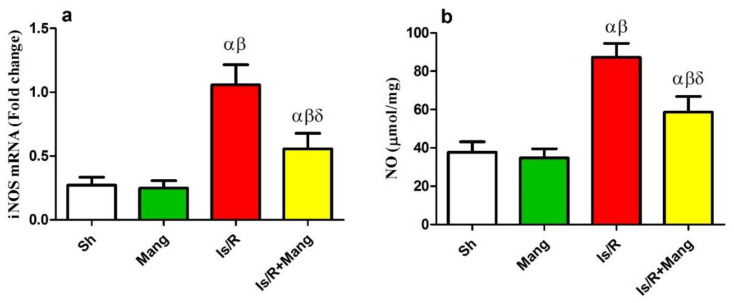
Mang’s (20 mg/kg, i.p) impact on renal (**a**) iNOS mRNA protein expression and (**b**) NO content as nitrosative stress markers. Data were articulated as the mean (*n* = 7) ± SD, *p* value < 0.05 is significant. As compared with sham (α), Mang (β), and Is/R (δ) treated groups.

Figure 3 illuminates the upsurge of the inducible nitric oxide synthase (iNOS) mRNA expression and its byproduct; total nitric oxide (NOx) content by 3.9 and 2.3 folds, respectively, as compared to the Sh group. Nonetheless, pre-Mang administration retrogresses the Is/R consequences.

### 2.4. Impact of Mang on PPAR- γ, NF-κB p65, and TNF-α Expression

**Figure 4:**
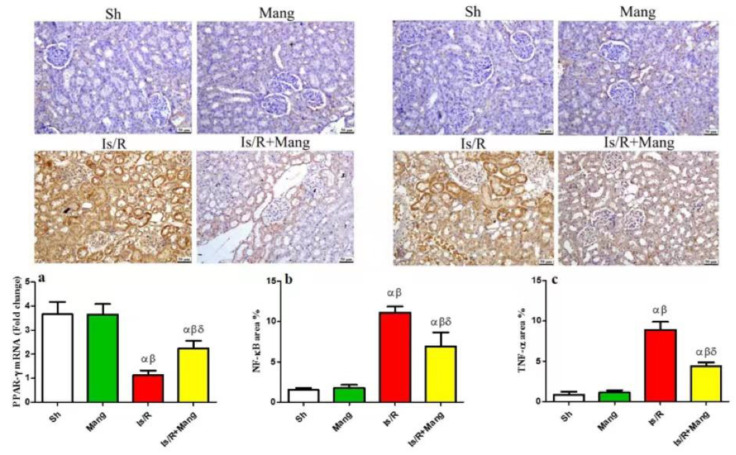
Mang’s (20 mg/kg, i.p) impact on renal (**a**) PPAR-γ mRNA protein expression as a signaling molecule, as well as IHC of (**b**) NF-κB p65, and (**c**) TNF-α protein expression as inflammatory parameters. Data were articulated as the mean (*n* = 7) ± SD, *p* value < 0.05 is significant. As compared with sham (α), Mang (β), and Is/R (δ) treated groups.

As presented (Figure 4), the Is/R lesion has downsized (a) PPAR-γ mRNA expression and up-regulated immunohistochemistry (IHC) expression of (b) NF-κB p65, and (c) tumor necrosis factor-alpha (TNF-α). Instead, the pre-administration of Mang faced these Is/R outcomes.

### 2.5. Impact of Mang on p38 MAPK and JNK Proteins Expression

**Figure 5:**
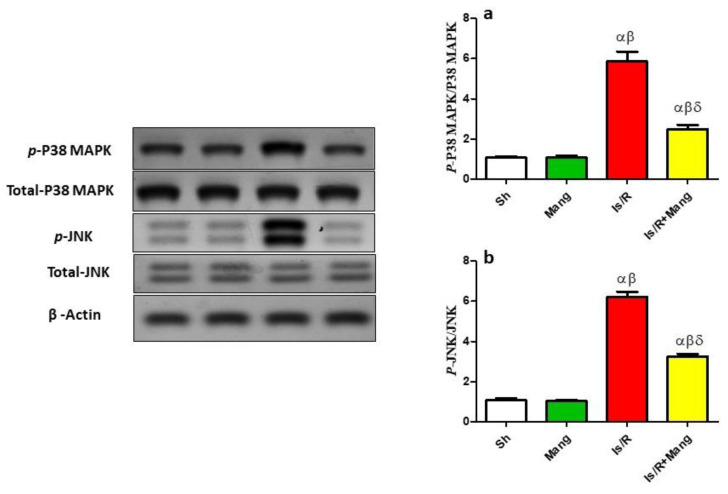
Mang’s (20 mg/kg, i.p) impact on renal (**a**) *p*-p38 MAPK, (**b**) *p*-JNK protein expression as signaling molecules. Data were articulated as the mean (*n* = 7) ± SD, *p* value < 0.05 is significant. As compared with sham (α), Mang (β), and Is/R (δ) treated groups.

As illustrated in Figure 5, the Is/R set disclosed an increase in the *p*-p38 MAPK and *p*-JNK protein expression (5.4 folds and 5.8 folds, respectively) as compared to Sh. Intriguingly, the Mang pretreatment was able to lessen these changes (Western supplementary as Figure S1c,d).

### 2.6. Impact of Mang on Apoptotic/Anti-Apoptotic Biomarkers

**Figure 6:**
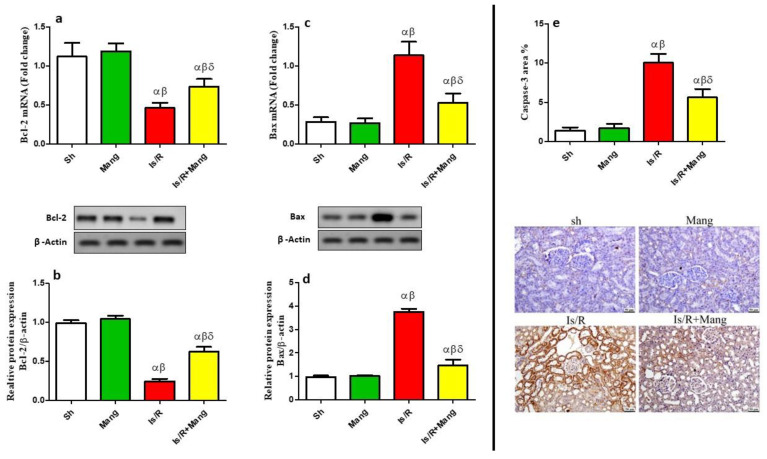
Mang’s (20 mg/kg, i.p) impact on (**a**) mRNA and (**b**) protein expression of renal Bcl-2, as well as (**c**) mRNA and (**d**) protein expression of renal Bax, and (**e**) IHC of caspase-3 protein. Data were articulated as the mean (*n* = 7) ± SD, *p* value < 0.05 is significant. As compared with sham (α), Mang (β), and Is/R (δ) treated groups.

As disclosed in Figure 6, apoptosis was triggered in Is/R kidney cell death as signified by the downsizing of Bcl-2 and the augmentation in Bax and caspase-3 levels. Meanwhile, Mang’s anti-apoptotic action was signified by facing these results (Western supplementary as Figure S1e,f).

### 2.7. Impact of Mang on Biomarkers of Renal Function

**Figure 7:**
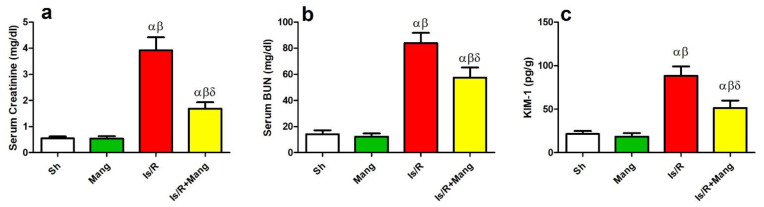
Mang’s (20 mg/kg, i.p) impact on serum (**a**) creatinine, and (**b**) BUN levels, and renal (**c**) KIM-1 content as renal function biomarkers. Data were articulated as the mean (*n* = 7) ± SD, *p* value < 0.05 is significant. As compared with sham (α), Mang (β), and Is/R (δ) treated groups.

Figure 7 revealed the surrogate markers of kidney injury, including serum (a) creatinine, (b) blood urea nitrogen (BUN), and (c) kidney injury molecule-1 (KIM-1) were amplified (7.2, 5.9, and 4.1 folds, respectively) in the Is/R group compared to Sh. Contrariwise, Mang pretreatment before Is/R diminished these parameters. 

### 2.8. Impact of Mang on Renal Morphological Changes and Lesion Score

**Figure 8:**
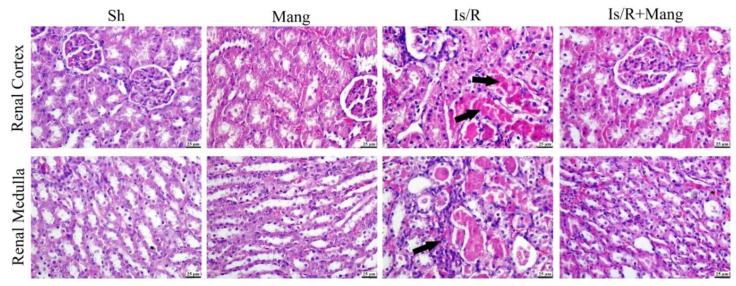
Mang’s (20 mg/kg, i.p) impact on renal histopathological alterations of renal cortex and renal medulla after bilateral renal Is/R (45 min/24 h) in Wistar rats.

**Figure 9:**
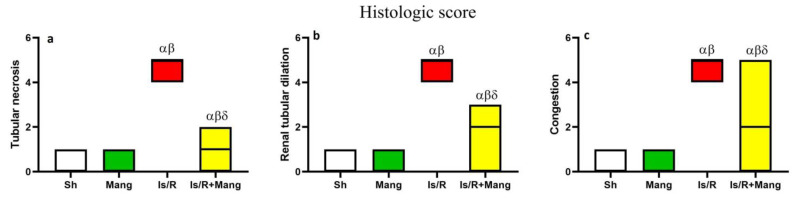
Mang’s (20 mg/kg, i.p) impact on renal (**a**) tubular necrosis, (**b**) tubular dilation, and (**c**) congestion as histologic scores after bilateral renal Is/R (45 min/24 h) in Wistar rats. As compared with sham (α), Mang (β), and Is/R (δ) treated groups.

Kidney sections (Figure 8) from Sh and Mang groups appeared histologically normal, meanwhile, Is/R showed marked renal tubular necrosis that was associated with diffuse hemorrhage in both the renal cortex and medulla. Eosinophilic protein-rich cast was frequently detected in renal tubules with cystic dilatation. Congestion of renal vasculature was detected as well. Is/R+Mang group exhibited improvement as few renal tubules suffered from degenerative changes with congestion in renal capillaries, however, most of the examined sections were normal. A significant reduction in all estimated histologic scores (Figure 9) was observed in the Is/R+Mang group when compared to the Is/R group. No statistically significant difference was detected between the sham and Mang group.

### 2.9. Mang Molecular Docking of Nrf2, p38 MPAK, and JNK Proteins

Mang demonstrated moderate to auspicious binding affinities ranging from −3.51 to −7.08 kcal mol^−1^ (Table 1) on its target proteins with a variety of degrees of interactions.

**Figure 10:**
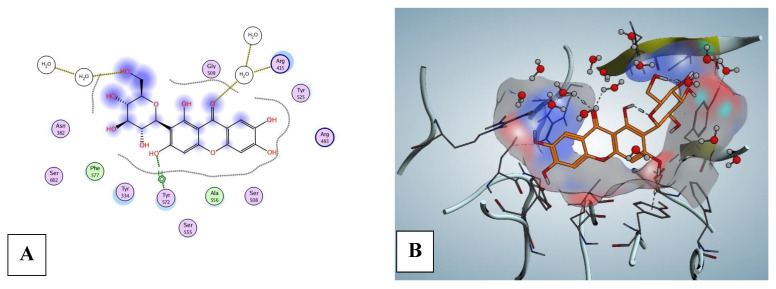
Nrf2 protien structure (PDB: 1X2R) (**A**) 2D and (**B**) 3D diagrams show the interaction of mangiferin (orange) in the binding pocket.

**Figure 11:**
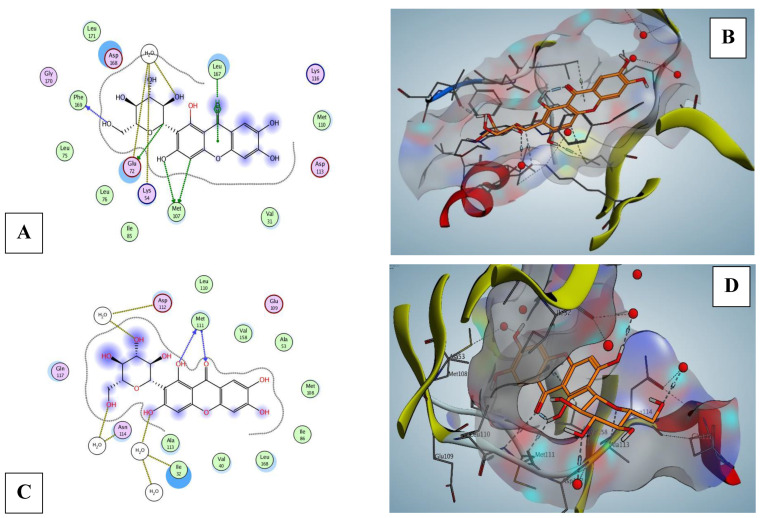
MAPK_13_ (p38-delta) (PDB: 4EYM) and JNK1 (PDB: 3ELJ) protiens, (**A**,**C**) 2D and (**B**,**D**) 3D diagrams respectively show the interaction of Mang (orange) in the binding pocket.

In Figure 10, Mang exhibited interactions in Nrf2 protein with 3 H-bonds between HOH228, HOH57, and TYR572 (H-pi). On the other hand, Mang was fitted on MAPK_8_ (JNK1) and p38 MAPK proteins as an inhibitor. Mang was fitted on MAPK_8_ (JNK1, −7.08 kcal mol^−1^ binding affinities) and interacts by five H-bonds, mainly between the hydroxyl groups and MET111 (Figure 11). Mang was fitted on p38 MAPK proteins; beta, delta, and alpha (from −3.51 to −6.46 kcal mol^−1^ binding affinities). Best in silico activity was recorded on MAPK_13_ (p38-delta) with a binding affinity −6.46 kcal mol^−1^ and five H-bonds, primarily between the hydroxyl groups and MET107, GLU72, PHE169, HOH557, moreover, LEU167 (pi-H interaction) (Figure 11). Molecular docking of MAPK_11_, MAPK_13_ (PDB ID: 4EYJ), and MAPK_14_ were supplied in Supplementary Materials as Figure S2a,b.

## 3. Discussion

The current work clarifies the Mang defense activities against renal damage caused by the Is/R model by validating varied mechanistic elements. The triggering of the transcription factor Nrf2, which reduced the os load and the inflammatory cascades, is crucial to the protective effects of Mang against renal assault. Another important mechanism that clarified the glucosylxanthone drug’s beneficial protective effect was the apparent activation of PPAR-**γ**, as well as the inhibition of *p*-p38 MAPK and *p*-JNK that leads to the inhibition of the strategic transcription factor, NF-κB. Additionally, apoptosis was recognized as a distinctive event connected to the renal Is/R lesion through Bcl-2, Bax, and caspase-3 inspection, and they were successfully modified upon Mang use. As a mirror image to these positive sequels, histopathological results and kidney function biomarkers were in the same milieu.

The existing signal transduction findings for the Nrf2/HO-1 cue revealed that Is/R had notably declined the mRNA/protein expression of Nrf2 and HO-1 protein expression. Our results also revealed that there was an observed state of os after Is/R which was confirmed by an increase in the renal MDA and a decrease in the GSH content. The previous results were in line with previous studies [3,15,16,17,18]. This highlights the role of os induced by Is/R in renal tissue injury. In the same milieu, Mang treated the group at a dose of 20 mg/kg, i.p elevated the renal Nrf2 at different levels and HO-1 expression to suppress the os state and prevent further renal tissue damage. The antioxidant/renoprotective role of Nrf2/HO-1 had been highlighted previously [19]. Moreover, Mang at the same dose used in our study tackled Is/R induced-gastric ulcers by elevating Nrf2 and HO-1 mRNA levels [20]. Additionally, it has been reported that upregulation in the Nrf2 can stimulate γ-glutamylcysteine ligase which subsequently leads to an increase in GSH synthesis [20]. In addition, HO-1′s ability to eventually produce bilirubin from heme contributes to its antioxidant properties, which prevent lipid peroxidation. To our knowledge, this result represents the first demonstration of the protective role of Mang in the renal Is/R model via the antioxidant Nrf2/HO-1 pathway. 

Mang extended its activities to include the nitrosative stress as well as restoring the deranged redox status. Mang challenged the Is/R upshots in the iNOS/NOx levels, which generates the peroxynitrite anion-boosting os mediated lesion. Such an outcome is consistent with numerous earlier studies [21,22], pointing to a potential new tool for the Mang protective effect.

On the other hand, several studies highlighted the reno-protective role of PPAR-γ agonists in renal Is/R [7,23,24]. Their reno-protective effect has been explained to occur due to the ability of PPAR-γ agonists to inhibit NF-κB, therefore inhibiting the production of inflammatory cytokines and apoptotic factors [23]. In our study, we illustrated that there was a considerable reduction in the PPAR-γ mRNA level in the Is/R group as compared to normal rats. Furthermore, pretreatment of rats with Mang caused a significant elevation of the PPAR-γ mRNA level. Our results were in line with previous studies that showed a significant reduction in the PPAR-γ mRNA and content after Is/R, while Mang treatment reverted that by elevating the PPAR-γ level in induced gastric ulcers and intestinal injury models [20,25]. Moreover, another recent study highlighted the reno-protective role of Mang against methotrexate-induced toxicity and stated that this was due to its ability to elevate the PPAR-γ mRNA level [22].

Furthermore, this study revealed that after Is/R the immunohistochemical renal expression of NF-κB, TNF-α, and caspase-3 was elevated. In addition, there was a substantial rise in the mRNA and protein level of Bax, and a reduction in that of Bcl-2. These results were in line with a previous study [26]. We can suggest that Is/R triggered the state of os that led to a decrease in PPAR-γ mRNA level, which subsequently elevated the expression of NF-κB. The NF-κB increased the transcription of inflammatory cytokines (TNF-α) and apoptotic parameters such as caspase-3 and Bax while decreasing the anti-apoptotic Bcl-2. The Mang pretreatment elevated the mRNA level of PPAR-γ and mitigated the renal NF-κB immunohistochemical expression, which was accompanied by a decrease in the inflammatory and apoptotic parameters. These findings were confirmed previously in a study that assessed the reno-protective effect of Mang in methotrexate-induced renal injury [22].

Additionally, we found that the renal expression of either *p*-p38 MAPK or *p*-JNK was elevated in the Is/R group as compared to the normal one [27,28]. It was suggested that the os state that accompanies Is/R leads to the activation of *p*-p38 MAPK and *p*-JNK that subsequently activates NF-κB to amplify inflammatory cytokines transcription such as TNF-α and the apoptotic proteins [9,27]. Furthermore, it had been previously proven that inhibitors of p38 and JNK possessed the capability to reduce the apoptosis process [10]. Additionally, the role of JNK in inhibiting the production of anti-apoptotic protein (Bcl-2) had been mentioned previously [29]. Mang treatment decreased the renal tissue expression of both *p*-p38 MAPK and *p*-JNK which had been confirmed by various studies [30,31,32].

To the best of our knowledge, the Mang molecular docking on the aforementioned proteins was done for the first time in the in-silico investigation, showing an encouraging binding affinity that was consistent with the findings of the in vivo study.

Consequently, to the abovementioned changes, it was found that there was an evident deterioration in the kidney function in the Is/R group as compared to normal rats. This was shown by observing a critical amplification in the serum creatinine, BUN, and KIM-1 levels in the aforementioned group, additionally, it was confirmed by the histopathological deterioration. These outcomes were fortified by various studies which attributed kidney injury after Is/R due to os, inflammatory, and apoptotic status [18,33,34]. Pretreatment of rats with Mang before Is/R caused a significant reduction in the previously mentioned elevated kidney function parameters that were mirrored in the histopathological results, highlighting its reno-protective role that had been confirmed previously [14].

## 4. Material and Methods

### 4.1. Animals 

Male Wistar rats (210–260 g) acquired from the animal house of the Research Institute of Ophthalmology (Giza, Egypt) and maintained on October 6 University (O6U) animal house under controlled conditions (22 ± 2 °C, 12-h light/dark cycle). The present protocol follows NIH guidelines and O6U Research Ethics Committee approved the current protocol (Approval number: PRE-Ph-2201025).

### 4.2. Experimental Design

Animals (*n* = 7/group) were arbitrarily distributed into four groups. The first group was Sh one while the second group received Mang (20 mg/kg, i.p; Sigma-Aldrich, St. Louis, MO, USA). The third group was the Is/R and the last group was administered Mang (20 mg/kg, i.p) following the work of Awny et al. [20]. Except for the first and second groups, all the animals received saline (the vehicle) or Mang for a week before being subjected to Is/R (45 min/24 h).

### 4.3. Renal Ischemia/Reperfusion Induction

Is/R was accomplished as described by Mansour et al. [35]. Concisely, the rats were anaesthetized (xylazine/ketamine; 10/75 mg/kg, i.p) and then placed supine, after which a midline abdominal incision was operated and both renal pedicles were occluded using a microvascular clamp for 45 min. After that, the clamp was carefully detached, and the incision was sutured to allow reperfusion for 24 h. 

### 4.4. Sample Preparation

Wistar rats were reanesthetized following the completion of the reperfusion phase, and blood was drawn from the abdominal aorta to assess kidney function. After blood collection, the rats were euthanized, and both kidneys harvested. The first kidney was washed, weighed, and homogenized in ice-cold saline (stored immediately at −80 °C for the biochemical and ELISA assay). The second kidney (*n* = 3 rats/group) was preserved in 10% buffered formalin for morphological and IHC inspection. The remaining kidneys were cut off into two segments; the first segment was homogenized for western blot analysis using lysis buffer and the last fraction was flooded in RNA later solution for relative gene expression assay via quantitative real-time polymerase chain reaction (qRT-PCR).

### 4.5. Biochemical and ELISA Assay

The serum levels of creatinine and BUN as well as the renal content of glutathione (GSH), MDA, and NOx were estimated using Biodiagnostic kits, Cairo, Egypt (CR 12 50, UR 21 10, GR 25 11, MD 25 29, and NO 25 33, respectively). Meanwhile, the serum KIM-1 (SEA785Ra) ELISA kit was purchased from USCN (Wuhan, China). According to the guidelines of the manufacturers, all steps were conducted.

### 4.6. Western Blot

This technique was used for determination of protein expression of Nrf2, HO-1, *p*Thr180/Tyr182-p38 MAPK, *p*Thr183/Tyr185-JNK, Bax, and Bcl-2 in renal tissues [36,37]. Briefly, the renal tissues were washed, homogenized (pre-cold lysis buffer), and supplemented with protease/phosphatase inhibitor cocktails (Sigma, St. Louis, MO, USA). Total proteins determination was performed colorimetrically. Thirty µg of protein were incubated for 18–20 h at 4 °C with antibodies against Nrf2 (CAT # PA5-68817, Thermo Scientific, MA, USA), HO-1 (CAT.# ab13243, Abcam, CB, UK), JNK (CAT.# ab179461, Abcam, CB, UK), *p*-p38MAPK (CAT.# b170099, Abcam, CB, UK), Bax (CAT # MA5-14003, Thermo Scientific, MA, USA), Bcl-2 (CAT# PA5-27094, Thermo Scientific, Waltham, MA, USA), and β-actin (1:2500, # A5060, Sigma, St. Louis, MO, USA). After membrane washing, suitable secondary antibodies (Dako, Glostrup, Denmark) incubation was done. The Western Lightning Plus ECL Chemiluminescence Reagents (Perkin Elmer, Waltham, MA, USA) were mixed, applied, and the band signals/intensities were then captured via Chemi-Doc imager (Bio-Rad, Hercules, CA, USA).

### 4.7. qRT-PCR Technique

This technique was applied for the estimation of renal gene expression of PPAR-**γ**, Nrf2, iNOS, Bcl-2, Bax, and β-actin. For target gene relative expression determination, the 2^−ΔΔCT^ equation was used. Briefly, total RNA extraction was done using SV Total RNA Isolation System (Promega, Madison, WI, USA). Reverse transcription was done using an RT-PCR kit (Invitrogen, Carlsbad, CA, USA). SYBR Green PCR Master Mix was used for qPCR running (Applied Biosystems, Foster City, CA, USA). The primer sequences were documented in Table 2.

### 4.8. Renal Morphology and Lesion Score

Kidney tissue samples were kept in a 10% neutral buffered formalin. Afterward, the processing of the tissue and hematoxylin and eosin (H&E) staining protocol was followed to prepare stained tissue slides [38]. Tissue sections were inspected using Leica DM4B light microscope and images were captured using its attached digital camera (Leica, DMC 4500). On a scale ranging from 0 to 5, the severity of renal tubular necrosis, tubular dilatation, and renal vessel congestion was evaluated as previously described in Yang et al. [39].

### 4.9. Renal Immunohistochemistry

On adhesive slides, five µm tissue sections were cut for immune staining. Afterward, sections were rehydrated, subjected to heat-induced epitope retrieval, and incubated with primary antibodies (Mouse anti-NF-κB, anti-TNF-α, and anti-caspase-3 at a dilution of 1:150) for 2 hours at room temperature. After washing with TBS, tissue sections were blocked for endogenous peroxides for 30 minutes. Mouse/Rabbit Immuno-Detector DAB HRP kit was used as manufacturer instructions for color development [40]. Positive expression was quantified as area percent using Las-X (Leica software).

### 4.10. Molecular Docking

Nrf2, p38 MPAK, and JNK protein structures were downloaded from the protein data bank. Structure preparation and docking validation (low binding energy score and small RMSD) proceeded as a default setting on MOE 2016.10. Mangiferin-receptor interactions were evaluated on the validated protocol. Graphical (2D and 3D) diagrams showed the binding mode of Mang in the binding pocket were created.

### 4.11. Statistical Analysis

The one-way analysis of variance (ANOVA) with post-test Tukey’s multiple comparisons was performed. Meanwhile, histologic scores were presented as the median and analyzed using the Kruskal–Wallis/Mann–Whitney U test. A *p* value < 0.05 was considered significant. The data were shown as mean ± standard deviation (SD). Figures drawing and statistical analysis were accomplished by using version 5.01 GraphPad Prism (San Diego, CA, USA).

## 5. Conclusions

Finally, we concluded that Mang possessed a reno-protective effect against Is/R via mitigating the os state by activating Nrf2/HO-1 milieu. Furthermore, it had the capability of modulating PPAR-γ/NF-κB, and MAPK/JNK trajectories, inhibiting the inflammatory and apoptotic conditions. This highlights the suggested intracellular signaling milieus targeted by Mang offering a reno-protective effect against ischemia/reperfusion. 

## Figures and Tables

**Table 1:** Docked conformations of mangiferin with the target proteins.

Proteins	Energy Score (kcal/mol)	No. of Interactions	H-Bonding Residues
Nrf2 protein			
1X2R	−5.56	3	HOH228, HOH57, TYR572 (H-pi)
MAPK_8_ (JNK1) protein			
3ELJ	−7.08	5	HOH510, MET111, HOH545, HOH475
MAPK (p38) proteins			
MAPK_11_ (p38-beta)			
3GC9	−5.56	5	ASP112, HOH574, ASN115, HOH480
MAPK_13_ (p38-delta)			
4EYJ	−3.51	7	GLU72, ARG68, HOH511, LEU167, HOH709 (pi-H)
4EYM	−6.46	6	MET107, GLU72, PHE169, HOH557, LEU167 (pi-H)
MAPK_14_ (p38-alpha)			
1OUY	−5.87	4	ALA111, SER154, LYS53
1OVE	−5.93	7	HOH1241, HOH1177, HOH1256, MET109, LYS53
1WBS	−4.64	6	LYS53, HOH2029, HOH2208, GLU71, HOH2205 (pi-H)

**Table 2:** Primer sequences used in the current study.

Primer	Primer Sequences	Accession Number
PPAR-γ	F: 5′-CAGGTACCAGGAGCAGAGCAAAGAGCTG-3′ R: 5′-GAGGTACCGCTCTGTGACAATCTGCCTGA-3′	NM_001145366.1
Nrf2	F: 5′-ATGGCC ACACTTTTCTGGAC-3′ R: 5′-AGATGTCAAGCGGGTCACTT-3′	NM_031789.2
iNOS	F: 5′-TGGGTGAAAGCGGTGTTCTT-3′ R: 5′-TAGCGCTTCCGACTTCCTTG-3′	S71597.1
Bcl-2	F:- 5′-GGGGATGACTTCTCTCGTCG-3′ R:- 5′-GACATCTCCCTGTTGACGCT-3′	NM_016993.2
Bax	F:- 5′-TCATGAAGACAGGGGCCTTT-3′ R:- 5′-CTGCAGCTCCATGTTGTTGT-3′	NM_017059.2
β-actin	F: 5′-CGT TGA CAT CCG TAA AGA CCT C-3′ R: 5′-TAG GAG CCA GGG CAG TAA TCT-3′	NM_031144.3

## Data Availability

Data is contained within the article.

## Supplementary Materials

The following supporting information can be downloaded at: https://www.mdpi.com/article/10.3390/ph16010006/s1, Figure S1: (a–g): Uncropped western blot; Figure S2: (a,b): Molecular docking supplementary.
