# Supplementary material for: Possible Implication of Nrf2, PPAR-γ and MAPKs Signaling in the Protective Role of Mangiferin against Renal Ischemia/Reperfusion in Rats
Source: Pharmaceuticals (Basel). 2022 Dec 21;16(1):6. doi: 10.3390/ph16010006 (PMC9863472; doi:10.3390/ph16010006)
Supplement: Supplementary file 1 [file pharmaceuticals-16-00006-s001.zip › pharmaceuticals-2070792-supplementary.pdf]

Figure S1. Uncropped western blot.

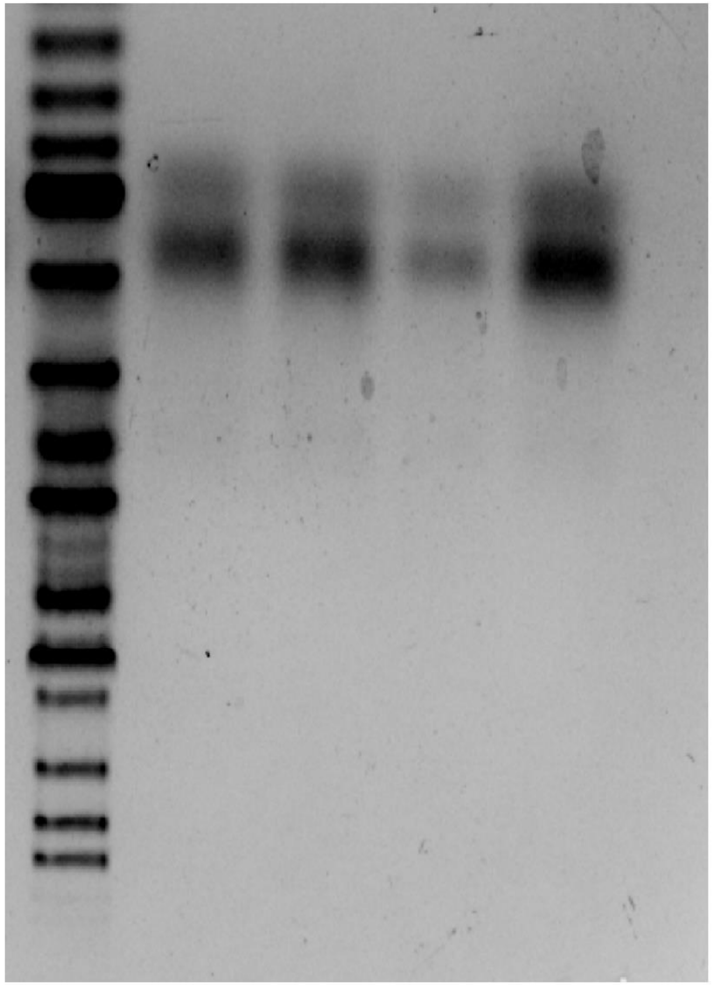

Figure S1 a: Nrf2

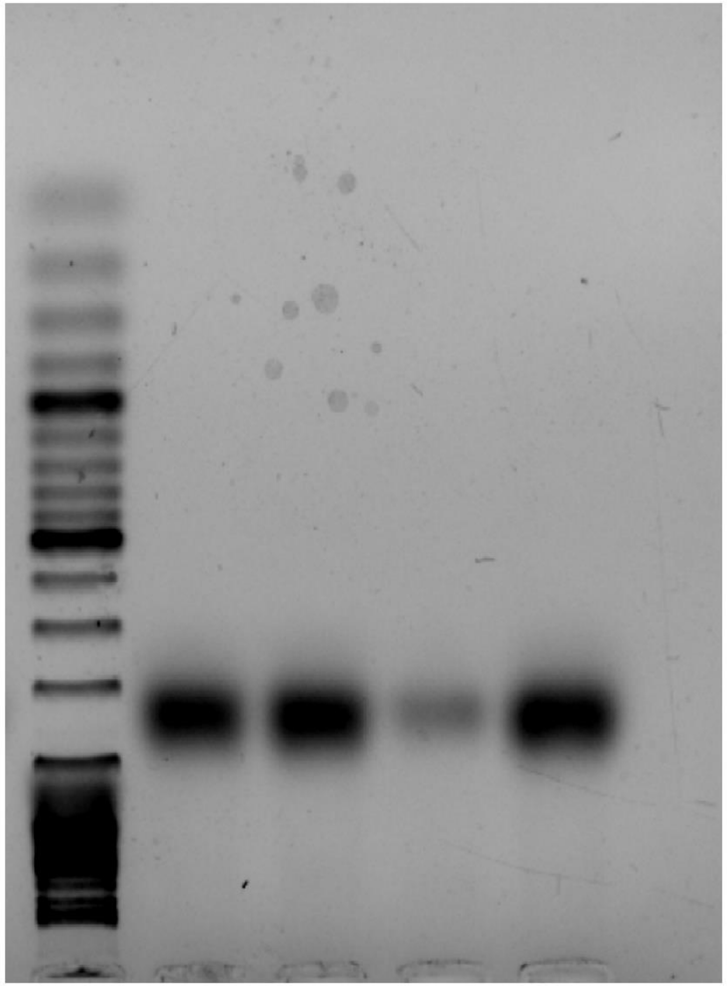

Figure S1 b: Ho-1

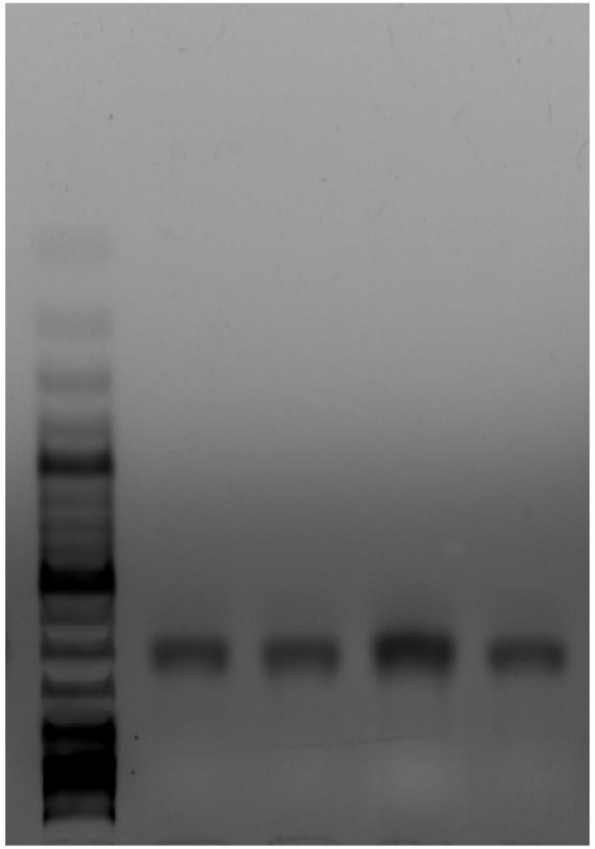

***P*-P38 MAPK**

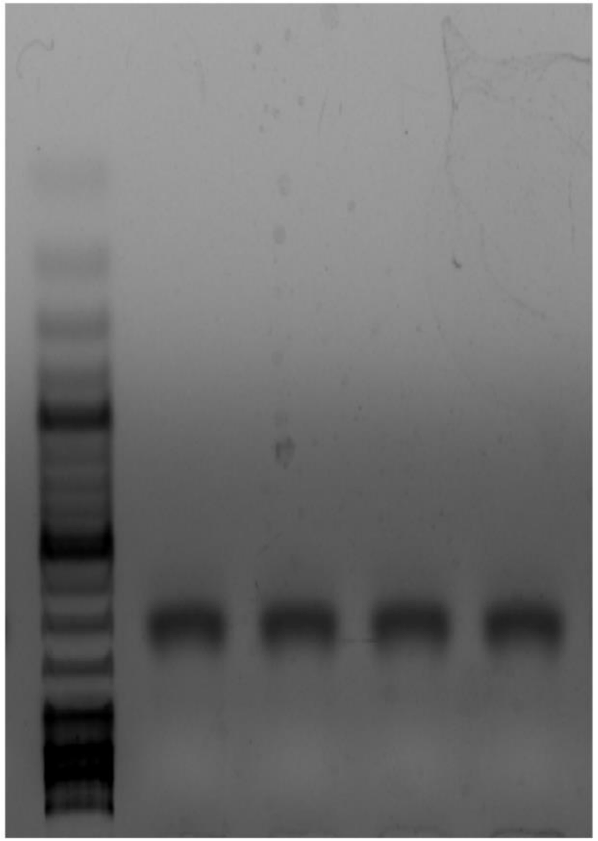

Total-P38 MAPK

Figure S1 c: *P*-P38 MAPK &Total-P38 MAPK

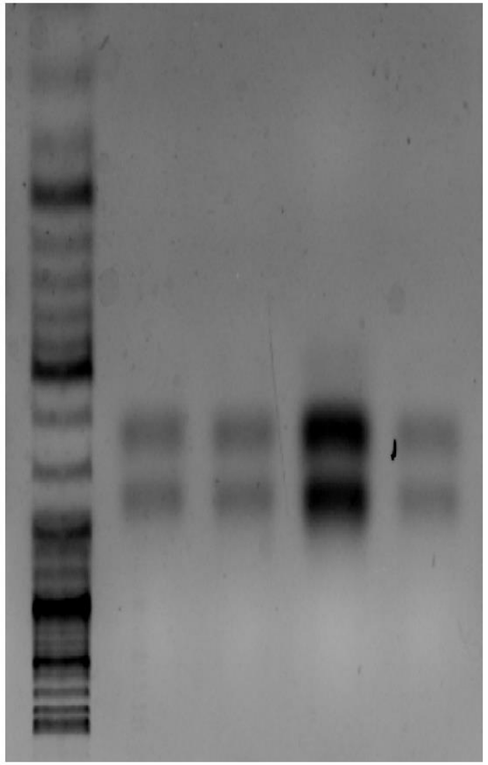

P-JNK

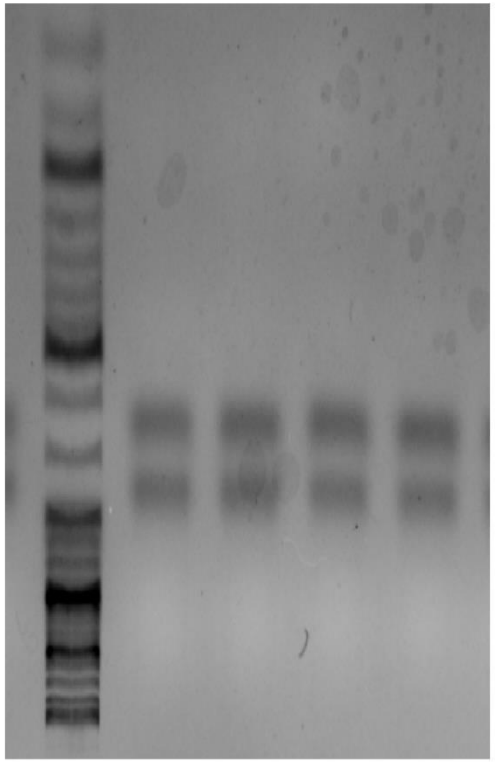

Total-JNK

Figure S1 d: *P*-JNK & Total-JNK

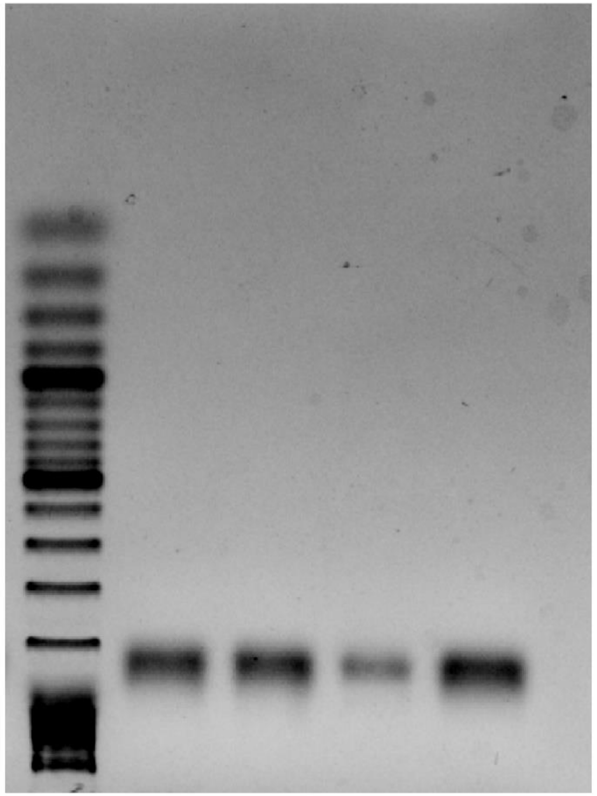

Figure S1 e: Bcl-2

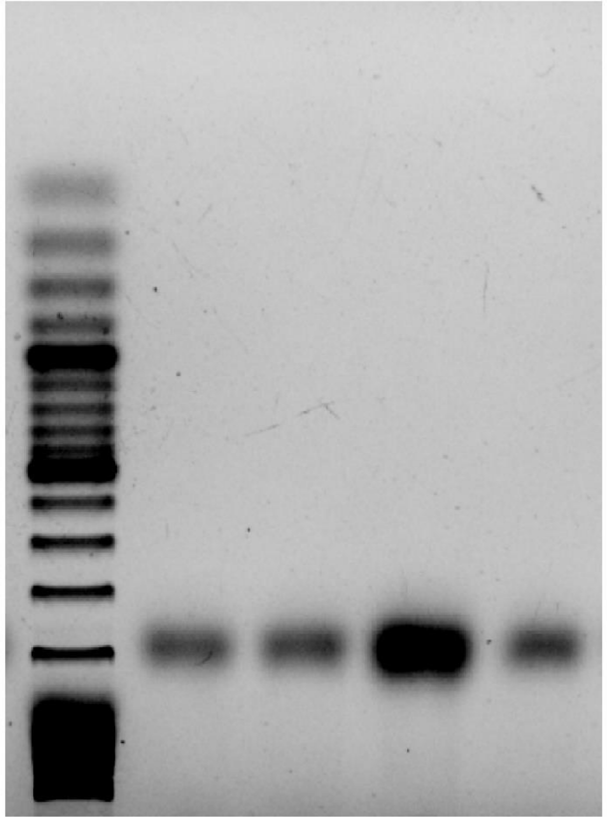

Figure S1 f: Bax

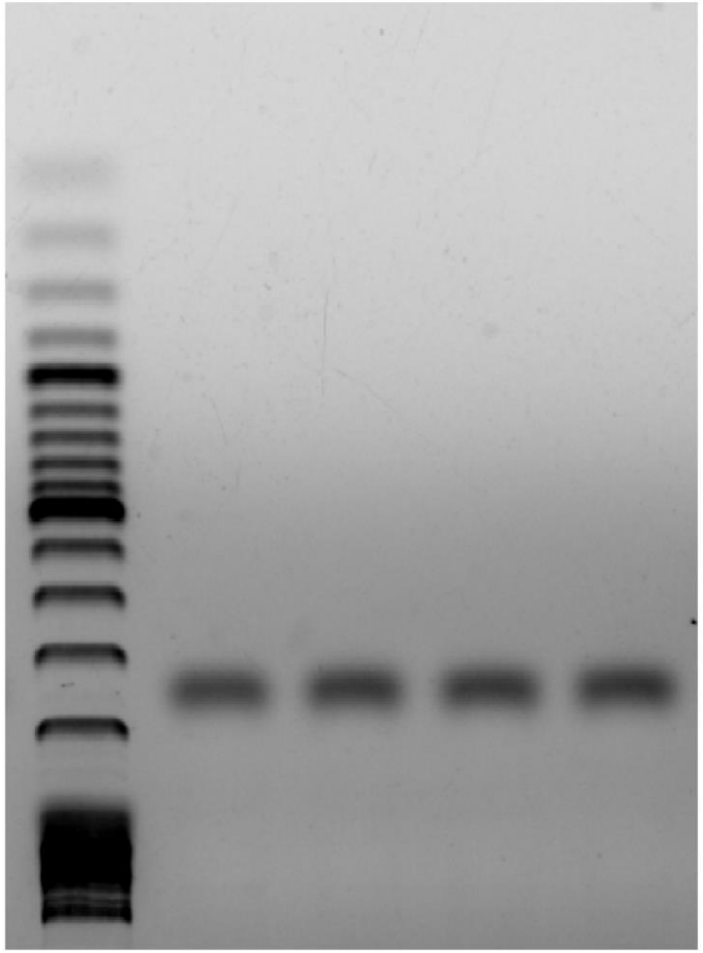

Figure S1 g: β-actin

Figure S2. Molecular docking supplementary

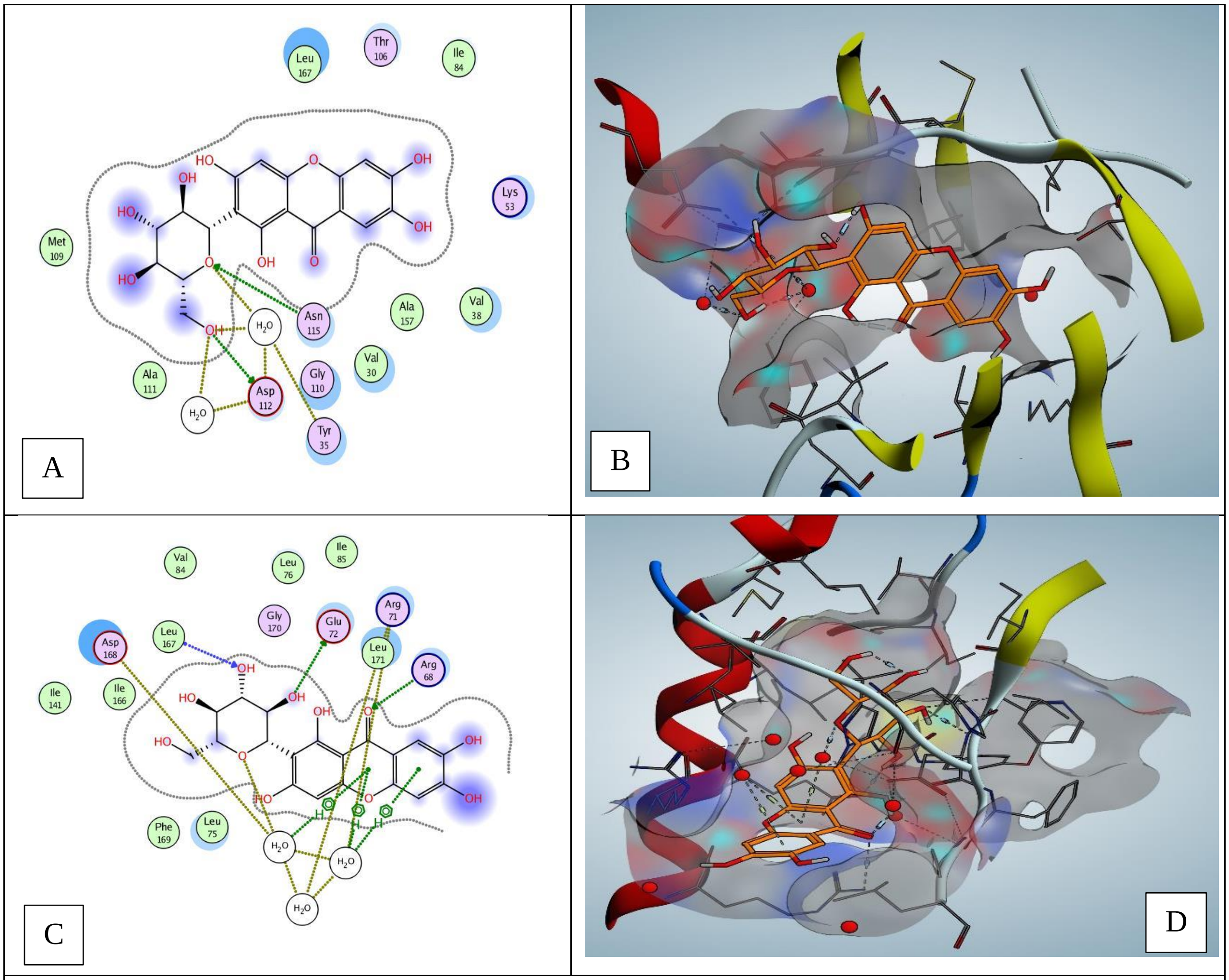

Figure S2 a. Crystal structure of **MAPK11 (p38- beta)** (PDB ID: **3GC9**) and **MAPK13 (p38-delta)** (PDB ID: **4EYJ**) complexes; (A, B) and (C, D) showed 2D and 3D diagrams respectively for binding mode of mangiferin (orange) in the binding pocket

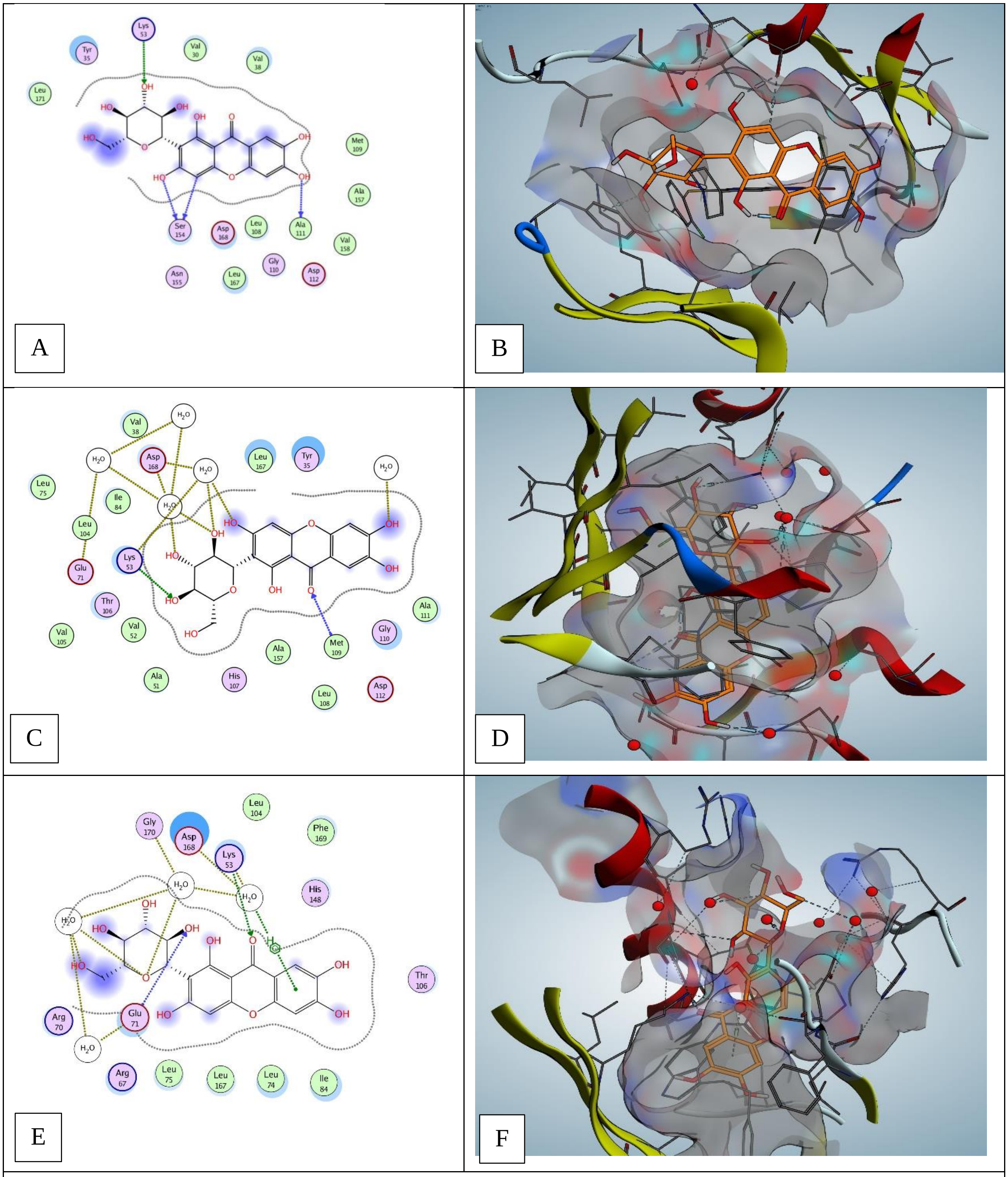

Figure S2 b. Crystal structure of **MAPK$_{14}$ (p38-alpha)** complex (PDB ID: **1OUY, 1OVE and 1WBS**); (A, B), (C, D), and (E, F) showed 2D and 3D diagrams respectively for binding mode of mangiferin (orange) in the binding pocket
